# The Effects of Single-Dose Rectal Midazolam Application on Postoperative Recovery, Sedation, and Analgesia in Children Given Caudal Anesthesia Plus Bupivacaine

**DOI:** 10.1155/2014/127548

**Published:** 2014-05-05

**Authors:** Sedat Saylan, Ahmet Eroglu, Davut Dohman

**Affiliations:** ^1^Department of Anesthesiology and Reanimation, Kanuni Education and Research Hospital, 61290 Trabzon, Turkey; ^2^Department of Anesthesiology and Reanimation, Faculty of Medicine, Karadeniz Technical University, 61080 Trabzon, Turkey

## Abstract

*Background*. This study aimed to compare the effects of rectal midazolam addition after applying bupivacaine and caudal anesthesia on postoperative analgesia time, the need for additional analgesics, postoperative recovery, and sedation and to find out its adverse effects in children having lower abdominal surgery. *Methods*. 40 children between 2 and 10 years of ASA I-II were randomized, and they received caudal anesthesia under general anesthesia. Patients underwent the application of caudal block in addition to saline and 1 mL/kg bupivacaine 0.25%. In the postoperative period, Group C (*n* = 20) was given 5 mL saline, and Group M (*n* = 20) was given 0.30 mg/kg rectal midazolam diluted with 5 mL saline. Sedation scale and postoperative pain scale (CHIPPS) of the patients were evaluated. The patients were observed for their analgesic need, first analgesic time, and adverse effects for 24 hours. *Results*. Demographic and hemodynamic data of the two groups did not differ. Postoperative sedation scores in both groups were significantly lower compared with the preoperative period. There was no significant difference between the groups in terms of sedation and sufficient analgesia. *Conclusions*. We conclude that caudal anesthesia provided sufficient analgesia in peroperative and postoperative periods, and rectal midazolam addition did not create any differences. This trial is registered with ClinicalTrials.gov NCT02127489.

## 1. Introduction

Pediatric patients need good recovery, sedation, and analgesia after surgery. Comfortable recovery period relaxes children, increases parent satisfaction, and provides doctor easier postoperative monitoring. As caudal analgesia is simple, reliable, and effective in postoperative analgesia, it is widely used in pediatric patients [1–3].

In postoperative period, less agitation and analgesic requirement is reported in children with caudal block after the induction of general anesthesia. However, postoperative analgesic effect may end early with a single dose of caudal local anesthetic and additional analgesia may be required. Researchers have started to use various drugs alone or in combination with other drugs in a caudal way to ensure effective and long-lasting analgesia. Rectal midazolam is reported to provide good sedation and inhibit agitation in pediatric patients [4, 5].

In this study, a noninvasive application of 0.30 mg/kg rectal midazolam was added to caudal anesthesia providing effective postoperative analgesia. We aimed to find out the effects of 0.30 mg/kg rectal midazolam addition after caudal anesthesia with 0.25% bupivacaine on postoperative analgesia time, the need for additional analgesics, postoperative recovery, and sedation in children. The adverse effects were also studied.

## 2. Methods

After the approval of local ethics committee and informed parental consent, 40 children (ASA physical status I-II; age 2–10 years) scheduled for lower abdominal surgery were randomly allocated to two groups of twenty each. Children with significant respiratory system, circulatory system, and liver and kidney function disorder and history of allergy to the drugs to be studied, those who received analgesic medication before the operation, and those for whom caudal anesthesia is contraindicated clinically were excluded from the study. Control Group (Group C) received caudal anesthesia with bupivacaine, and Group Midazolam (Group M) received rectal midazolam following caudal anesthesia with bupivacaine.

No premedication was given to the patients. Noninvasive arterial blood pressure, pulse, oxygen saturation, and temperature were recorded preoperatively with 4-point sedation scale.

Sedation scale for preoperative and postoperative assessment (1–4 points) represents the following states:

1. calm,
2. not quiet but easy to calm,
3. not easy to calm, moderately agitated, or restless,
4. angry, excited, or disoriented.

After monitoring the ECG, noninvasive blood pressure, and peripheral oxygen saturation (Drager Cato, Germany) and measuring the temperature of the patients taken to the operating room, sevoflurane was maintained with 50% nitrous oxide in O_2_, with induction starting at a concentration of 8% and fall by 2-3%. After obtaining adequate depth of anesthesia, venous cannulation (Mediflon, 22 G or 24 G, India) was performed. Patients were intubated after the use of 0.5 mg/kg atracurium for muscle relaxation. No analgesic drug was used. A warming blanket was used to prevent hypothermia during surgery. Patients in both groups were given lateral decubitus position for caudal block implementation and injected with 0.25% bupivacaine of 1 mL/kg volume diluted with saline slowly, after which the children were immediately turned supine. The maximum volume was 20 mL.

Mechanical ventilator (Drager Cato, Germany) was adjusted to end-tidal CO_2_ respiratory rate within the normal range and the volume continued at controlled ventilation. Anesthesia was maintained with 50% of O_2_, 50% of N_2_O, and 1.5–4% of sevoflurane. The depth of anesthesia, blood pressure, and heart rate values were maintained at ±20% of the initial values by changing the concentration of sevoflurane. None of the patients were given extra analgesic during anesthesia.

Systolic blood pressure (mmHg), heart rate (beats/min), peripheral oxygen saturation (SpO_2_), and temperature values were recorded before the operation, after the application of caudal block, at 5, 10, 15, and 30 minutes during surgery, and after the operation. In addition, anesthesia and operation time was recorded. 5 mL of rectal applicators to be used after surgical intervention was prepared by another anesthetist who was not involved in the study. Group C was given 5 mL of saline, and Group M was given 0.30 mg/kg of midazolam diluted with 5 mL of saline rectally. The follow-up of the patients was done by an anesthetist who was not aware of the drug applied. Patients taken to postanesthesia care unit (PACU) were assessed by a sedation scale and a postoperative pain scale (CHIPPS: The Children's and Infants' Postoperative Pain Scale). Patients with sedation scale over 2 were given 0.05–0.1 mg/kg midazolam for sedation. Patients with postoperative sedation scale over 3 were given 10–20 mg/kg of rectal paracetamol to stop the pain. Patients who were painless, calm, and with Aldrete Scale over 9 were sent to the related department.

### 2.1. Statistical Analysis

Conformity of the data obtained in measurements to the normal distribution was analyzed with Kolmogorov-Smirnov test. In the comparison of quantitative data of the two groups (control and midazolam groups), data that are in conformity with normal distribution were analyzed with Student's* t*-test, and data not conforming to normal distribution were analyzed with the Mann-Whitney* U* test. In the comparison of repeated measurements of intragroups, data that are in conformity with normal distribution in repeated measurements were analyzed with ANOVA (paired* t*-test as post hoc), and data not conforming to normal distribution were analyzed with Friedman test (Wilcoxon test as post hoc). Data obtained by measurements were given as mean ± standard deviation. The level of statistical significance was accepted as *P* < 0.05. For the studies with multiple statistical significance comparisons (post hoc), “0.05/comparison number” was used.

## 3. Results

40 patients were included in this study, 20 in each group. Caudal blockade was performed successfully at the first attempt in all children. No patient was excluded from the study. There were no significant differences between groups in demographic data (age, weight, and height) and duration of operation (*P* > 0.05) (Table 1).

Systolic arterial pressure (SAP), diastolic arterial pressure (DAP), and mean arterial pressure (MAP) measurements were shown in Table 2. In Group C, SAP at the 5th minute following caudal application was significantly lower than in Group M (*P* = 0.006). Timely changes of SAP, DAP, and MAP values decreased in Group M, which was statistically significant (*P* = 0.024, *P* < 0.005, and *P* < 0.005, resp.).

**Figure 1:**
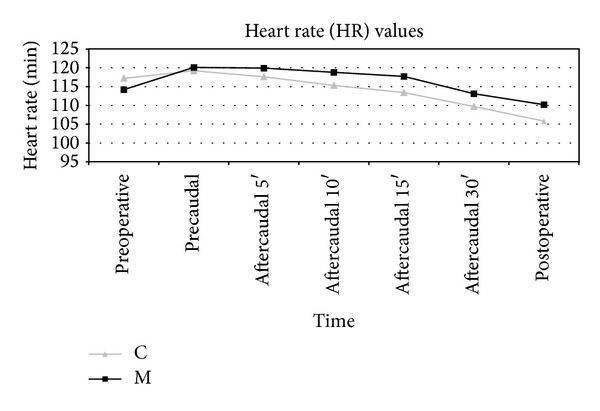
Heart rate values of patients (pc: precaudal, ac: after caudal).

Figure 1 shows the changes in heart rate of the groups. Timely changes of heart rate values in Group C and Group M were statistically significant (*P* = 0.034 and *P* = 0.015, resp.). Values of heart rate in both groups decreased in the postoperative period and after caudal application compared to preoperative and precaudal periods. However, there were no statistical differences between the groups.

**Figure 2:**
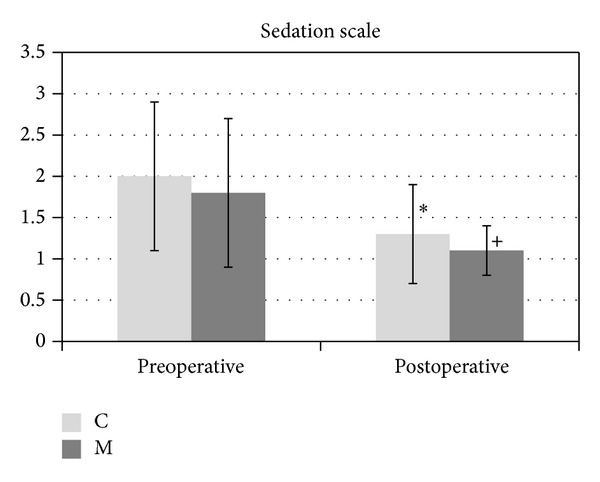
Sedation scale of the groups. *P = 0.021 when sedation scale in preoperative period is compared with that in postoperative period in Group C; ^+^ *P* = 0.005 when sedation scale in preoperative period is compared with that in postoperative period in Group M.

Sedation scores of the groups are shown in Figure 2. Timely changes in sedation scale of Group C and Group M decreased in postoperative period compared to preoperative period, which was statistically significant (*P* = 0.021 and *P* = 0.005, resp.). There was no statistically significant difference between the groups.

**Figure 3:**
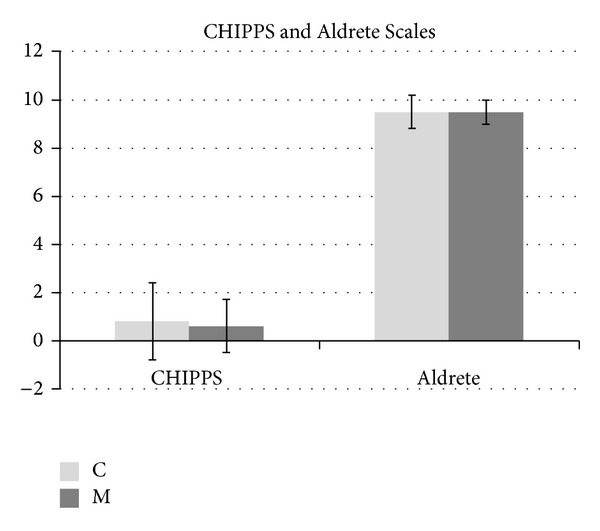
CHIPPS and Aldrete Scales of the groups.

There was no significant difference between CHIPPS and Aldrete Scales of the groups (Figure 3). As for side effects, agitation was observed in 1 patient and vomiting in another patient in Group C. Vomiting was observed in 3 patients and urinary retention in 1 patient in Group M (Table 3).

First analgesic time in Group C was 225.4 ± 324.5 min, while 7 patients did not require any analgesics for 24 hours. First analgesic time in Group M was 280.5 ± 300.4 min, while 9 patients did not require any analgesics for 24 hours. The groups did not show statistically significant difference in terms of first analgesic time (*P* = 0.581).

## 4. Discussion 

This study showed that caudal anesthesia provided sufficient analgesia in perioperative and postoperative periods; however, rectal midazolam addition did not create any differences. We used CHIPPS and Aldrete Scales for evaluation of sedation and recovery of patients. The most widely used scale is CHIPPS which is applied to children from 6 months to 12 years. This is a reliable, postoperative pain scoring system with five behavioral criteria: crying, facial expression, motor activity, posture, and leg movement [6–8]. Aldrete recovery score is a frequently used postanesthesia intensive care unit discharge scoring system with five main criteria: activity, respiration, circulation, consciousness, and colour. Patients with Aldrete score of 9 and above are safe to discharge. In our study, all of the patients were followed for 24 hours at the service for the first postoperative analgesic duration and complications (nausea, vomiting, motor block, hypotension, bradycardia, urinary retention, etc.).

The management of postoperative pain requires taking preventive measures. Regional anesthesia is often used together with general anesthesia in children [9, 10]. Caudal block has many characteristics such as early return to normal activity and excellent and fast analgesia in inguinal and genital areas. Various studies have shown that ACTH, immunoreactive beta-endorphin, ADH, cortisol, prolactin, and glucose levels are less affected after caudal block compared with general anesthesia [11, 12]. Caudal administration of bupivacaine is routinely used in pediatric patients undergoing genitourinary surgical procedures for improving postoperative pain relief [13, 14]. Although the effect of caudal analgesia with bupivacaine on postoperative pain relief has been extensively investigated in children, we did not find any study which included rectal midazolam for postoperative sedation. Therefore, we decided to study it.

Da Conceicao and Coelho [15] found that children given 0.375% caudal bupivacaine had first analgesic requirement 5 hours later. Different studies found different analgesia time in caudal application of bupivacaine, which may be due to differences in types of surgery, pain scoring systems, drug dosage and volume, analgesia time assessment methods, and family factor [15]. In our study, first analgesic requirement time was 225.4 ± 324.5 min in Group C, and 7 patients did not need any for 24 hours. It was 280.5 ± 300.4 min in Group M, and 9 patients did not need any for 24 hours. The groups did not show statistically significant difference in terms of the first analgesic time (*P* = 0.581).

Lower abdominal and genitourinary operations can lead to severe postoperative pain; therefore, it may cause agitation and restlessness in children [16–18]. Breschan et al. [19] reported that of 1845 single-dose caudal block applications with bupivacaine, only 2 patients developed total spinal block as a major complication who were intubated, ventilated, and extubated in 4 hours without any significant cardiovascular depression. Three patients had urinary retention in the same study. In our study, 1 patient had agitation and 1 patient had vomiting in Group C, and 3 patients had vomiting and 1 patient had urinary retention in Group M. Kanegaye et al. [5] reported that patient anxiety and unwanted movements can be frequently seen in pediatric operations even with effective local anesthesia, but adequate sedation procedure can improve technical results and increase patient, parental, and doctor satisfaction. They claimed that midazolam is the best sedative to have true state of sedation in children and provide required levels of security, anxiety, amnesia, quickness in movement after use, and pharmacological reversibility. Pediatric transmucosal midazolam application was first used by rectal, intranasal, and sublingual ways for sedation before anesthesia, took growing interest by researchers, and has become widely used. Kanegaye et al. [5] reported that midazolam is suitable for emergency pediatric patients and can be applied with a painless injection. In our study, we chose rectal midazolam which is known to be an effective, easily absorbable, and noninvasive method when administered rectally [4].

Kanegaye et al. [5] compared two different doses of rectal midazolam used at pediatric emergency department in terms of sedative efficacy and frequency of agitation. A group of patients taking cutaneous procedures were given 0.5 mg/kg standard dose, while another group was given 1 mg/kg rectal midazolam. They concluded that rectal midazolam improved sedation scores before operation, and 1 mg/kg midazolam was more effective. However, insufficient sedation was recorded in 27% to 50% of the patients with high doses, and 27% of the patients had prolonged agitation, which is a disadvantage for the use of rectal midazolam. They suggest that doctors should consider the possibility of dose-dependent inadequate sedation and agitation before choosing rectal midazolam for pediatric sedation [5]. In our study, agitation was observed in one patient in Group C, but none in Group M.

Mahajan et al. [20] evaluated the analgesic efficacy of midazolam and bupivacaine mixture in children undergoing genitourinary operations to relieve postoperative pain and also evaluated its side effects. They gave a single caudal injection of 0.5 mL/kg, 0.25% bupivacaine to the first group and 0.5 mL/kg, 0.25% bupivacaine together with 0.5 mL/kg, 50 microg/kg midazolam to the second group. They observed heart rate, arterial blood pressure, and oxygen saturation and assessed postoperative pain via an objective pain score at regular intervals for 12 hours. An analgesic was given when pain score was 4 or greater. Duration of analgesia as well as additional analgesic need was determined. They concluded that bupivacaine plus midazolam caudal application compared with single-dose bupivacaine provided longer postoperative analgesia without any side effects [20]. In our study, SAP, DAP, MAP, and heart rate decreased significantly in both groups after caudal anesthesia compared with preoperative period, but there was no significant difference between groups. Sedation scale of both groups decreased in postoperative period compared with preoperative period, but there was no statistically significant difference between groups. No differences occurred in CHIPPS and Aldrete scores of the groups. No differences were determined between groups in terms of adverse effects and first analgesic time.

In conclusion, we found in our study that the addition of 0.30 mg/kg rectal midazolam after caudal anesthesia with bupivacaine makes no difference in postoperative analgesia time, additional analgesia requirement, postoperative recovery, the effectiveness in ensuring sedation, and side effects in children having lower abdominal surgery, but caudal application of 0.25% bupivacaine provided effective and sufficient postoperative analgesia.

## Figures and Tables

**Table 1:** Demographic data, duration of operation, and types of operation.

	Group C (mean ± SD)	Group M (mean ± SD)	*P* value
Age (months)	62.9 ± 25.6	56.0 ± 24.9	0.359
Weight (kg)	19.2 ± 6.2	19.4 ± 5.3	0.857
Height (cm)	111.5 ± 13.8	110.7 ± 15.7	0.935
Duration of anesthesia (min)	60.5 ± 23.7	56.3 ± 16.6	0.515
Duration of operation (min)	45.5 ± 21.6	41.1 ± 15.1	0.806
Operation types			
Hypospadias	6	6	
Buried penis	1	3	
Cryptorchism	6	3	
Inguinal hernia	4	6	
Circumcision	2	1	
Hydrocele	1	1	

**Table 2:** Comparison of groups by SAP, DAP, and MAP.

	Group C (mean ± SD)	Group M (mean ± SD)	*P* value
SAP			
Preoperation	99.7 ± 24.3	106.8 ± 8.6	0.229
Before caudal	97.3 ± 6.6	100.3 ± 8.4	0.217
5th min after caudal	96.5 ± 6.7	103.0 ± 7.1	0.006*
10th min after caudal	97.0 ± 8.2	100.4 ± 6.2	0.141
15th min after caudal	95.6 ± 8.1	98.8 ± 7.0	0.190
30 min after caudal	94.2 ± 6.6	97.3 ± 8.0	0.212
Postoperation	103.0 ± 9.2	103.3 ± 6.4	0.921

DAP			
Preoperation	59.9 ± 10.2	56.9 ± 9.5	0.351
Before caudal	53.5 ± 6.2	53.7 ± 8.4	0.933
5th min after caudal	50.0 ± 8.4	53.1 ± 8.8	0.262
10th min after caudal	50.7 ± 8.5	50.6 ± 8.3	0.970
15th min after caudal	48.4 ± 8.1	48.1 ± 8.8	0.896
30 min after caudal	47.3 ± 10.3	44.7 ± 8.4	0.411
Postoperation	58.9 ± 12.4	53.7 ± 10.8	0.163

MAP			
Preoperation	73.7 ± 10.5	72.0 ± 9.4	0.581
Before caudal	66.9 ± 5.9	67.8 ± 8.2	0.709
5th min after caudal	64.0 ± 8.2	66.8 ± 8.0	0.289
10th min after caudal	63.5 ± 8.6	64.1 ± 7.9	0.805
15th min after caudal	62.2 ± 8.3	62.3 ± 7.9	0.969
30 min after caudal	60.2 ± 9.3	59.1 ± 7.7	0.699
Postoperation	72.9 ± 13.1	67.2 ± 9.5	0.127

*When SAP value at the 5th min after caudal in Group M is compared with that in Group C.

**Table 3:** Side effects in patients in both groups.

Side effects	Group C		Group M	
	*n*	%	*n*	%
Agitation	1	5.0	—	—
Vomiting	1	5.0	3	15.0
Urinary retention	—	—	1	5.0
No side effect	18	90.0	16	80.0
